# Intravenous Immunoglobulin Inhibits Liver Cancer Progression by Promoting p38MAPK-Associated Apoptosis

**DOI:** 10.1155/2022/1300989

**Published:** 2022-07-14

**Authors:** Fengjie Xu, Runzhui Lin, Jianrui Liu, Zeming Chen, Hua Zhuo, Xingmu Liu

**Affiliations:** ^1^Shantou University Medical College, Shantou, China; ^2^Second Affiliated Hospital of Shantou University Medical College, Shantou, China

## Abstract

**Objective:**

The aim of this study is to explore the effect of intravenous immunoglobulin (IVIG) on the development of rat hepatocellular carcinoma and its possible molecular mechanism.

**Methods:**

Sixty adult male Sprague-Dawley (SD) rats were randomly divided into three groups: control, diethylnitrosamine(DEN) + normal saline(NS), and DEN + IVIG groups, with 20 rats in each group. The  rats in the DEN + NS group and DEN + IVIG group were given DEN 0.2 g/kg intraperitoneal injection once on day 1 and then 0.05% DEN aqueous solution in drinking water to establish a rat liver cancer model. Immunoglobulin (IgG) was injected intraperitoneally into the DEN + IVIG group twice a week at the dose of 100 mg/kg, and saline was administered intraperitoneally into the control group at a 50 mg/kg dosage. The body weight of each group of rats was recorded twice a week. All treatments were maintained continuously for 12 weeks. After the intervention, the liver function indexes of rats were measured by a fully automated biochemical analysis instrument. The liver histopathology was observed by hematoxylin-eosin(HE) staining. Immunohistochemistry was used to detect c-myc protein expression, and Western blotting was used to determine p38MAPK and p-p38MAPK protein expressions, as well as apoptosis-related proteins such as Bcl-2, Bax, and cleaved caspase-3.

**Results:**

Compared with the rats in the DEN + NS group, rats in the DEN + IVIG group showed substantially higher body mass (*P* < 0.05), higher survival rate (*P* < 0.05), and lower liver function indexes (*P* < 0.05). Few focal necrosis of cancer cells and few nuclear division were observed in the rats in the DEN + IVIG group. The rats in the DEN + NS group showed lamellar necrosis of cancer foci, destruction of normal liver lobular structure, and hepatocellular carcinoma cells. Immunohistochemical analysis results revealed that the expression of c-myc was reduced in the DEN + IVIG group (*P* < 0.05), and Western blotting confirmed that the Bcl-2 expression was decreased (*P* < 0.05), while Bax, p38 MAPK, p-p38 MAPK, and cleaved caspase-3 protein expressions were increased (*P* < 0.05).

**Conclusion:**

IVIG prophylactic injection can delay tumor development and induce apoptosis in primary hepatocellular carcinoma in rats. The mechanism is connected to the activation of the p38MAPK signaling pathway by upregulating the level of cleaved caspase-3 and Bax proteins while downregulating the level of Bcl-2 and c-myc proteins.

## 1. Introduction

Hepatocellular carcinoma (HCC) is highly malignant, insidious, and rapid in onset and progression and is prone to metastasis. HCC ranks the second highest mortality causal tumor among malignant tumors [1, 2], and the prognosis is poor. Most patients with hepatocellular carcinoma are diagnosed in the middle and advanced stages. Therefore, most patients have lost the opportunity for surgery. In addition, even with radical resection, 60%–70% of patients have a metastatic tumor within 5 years, and the rate of metastatic recurrence is high with local treatment [3, 4]. Immunotherapy has been applied to treat hepatocellular carcinoma and has shown some efficacy. However, its mechanisms of action remain unknown.

Intravenous immunoglobulin (IVIG) is immunoglobulins (IgG) with antibody activity and has a broad-spectrum of antibacterial and antiviral effects. According to previous studies, IVIG can effectively inhibit proliferation, invasion, and metastasis in tumor cell lines, tumor animal models, and tumor patients [5, 6]. However, the antitumor mechanism of IVIG remains unclear. This research aims to investigate the mechanism of IVIG-induced apoptosis and apoptosis-related genes in rat hepatocellular carcinoma. We explore the p38MAPK signaling pathway in IVIG-mediated apoptosis and inhibition of tumor development and metastasis in a rat hepatocellular carcinoma model. This study reveals the mechanism of IVIG in inhibiting hepatocarcinogenesis and development.

## 2. Material and Methods

### 2.1. Animals

Sixty male Sprague-Dawley (SD) rats (SPF class, weight 180–210 g) were purchased from Shantou University School of Medicine's Experimental Animal Center. The animals were kept in a 12 h/12 h alternating light and dark environment with a temperature of 22 ± 2°C and a relative humidity of 50–60%. Food and water were provided ad libitum. The Ethics Committee at Shantou University School of Medicine approved the proposal to all animal manipulations. (Number: SUMC2019-185).

### 2.2. Experimental Reagents

Total IgG was purified from SD rat serum using agarose protein G (Beyotime, Jiangsu, China). Pierce bicinchoninic acid(BCA) protein kit was used to detect IgG concentration (Thermo Fisher Scientific Inc, Waltham, US). The following chemicals were also used: DEN (Item No. N-0756, purchased from Sigma). Anti-p38 mitogen-activated protein kinase (p38 MAPK) antibody (8690T, CST); p-p38MAPK (4511T, CST); Bcl-2 (AF6139, Affinity); Bax (A00183, Affinity); *β*-actin (BM0627, BOSTER); and hematoxylin-eosin (HE, G1120, Sole Bauer).

### 2.3. Experimental Design

Sixty adult male Sprague-Dawley (SD) rats, weighing 200 ± 10 g and aged 6–8 weeks, were randomly divided into three groups: control, DEN + NS, and DEN + IVIG groups, 20 rats in each group. A diethylnitrosamine(DEN) solution was arranged at the concentration of 10 mg/ml in phosphate buffered solution(PBS) and administered to the DEN + NS and DEN + IVIG rats at a one-time dose of 200 mg/kg intraperitoneally on day 1. Then, all DEN-treated rats were given 0.05% DEN aqueous solution to drink for 12 weeks to induce hepatocellular carcinoma. For treatment, control rats were given saline 10 ml/kg body weight intraperitoneally once, DEN + IVIG rats were injected with IgG at a dosage of 100 mg/kg intraperitoneally twice a week for 12 weeks, and DEN + NS rats were injected at the same dosage of saline intraperitoneally for 12 weeks. During the experiment, the animals were observed daily for mental status, diet, and coat change, and the body weight change of rats were tracked weekly. At the end of the 14th week, blood was drawn from the heart to isolate serum, and the livers were formalin-fixed and paraffin-embedded.

### 2.4. Histopathological Examination

Liver tissues from each group of rats were soaked in 10% formalin fixative for 24 h and embedded in paraffin subsequently, and cut into 5-*μ*m-thick sections for routine HE staining and observed by light microscopy. The rat liver tumor tissues were evaluated pathologically using the Edmondson grading [7].

### 2.5. Serum Biochemical Evaluation

Blood was obtained from the rats by cardiac puncture and serum samples were isolated. The serum levels of ALT(alanine aminotransferase), AST(aspartate transaminase), ALP(alkaline phosphatase), and GGT(gamma glutamyl transpeptidase) were estimated according to the manufacturers' instructions of the ELISA kits (EK1573, BC1555, BC1565, AB241029, Solarbio, Beijing, China).

### 2.6. Immunohistochemistry

Rat liver sections were dewaxed, hydrated with a gradient series of alcohol, then rinsed with PBS, treated with 3% hydrogen peroxide at the room temperature of 18–20°C for 5–10 min, and then blocked with 5%–10% normal goat serum for 30 min. Primary antibodies were diluted in PBS and incubated on sections overnight at 4°C. After that, the slices were then rinsed three times with PBS and incubated at 37°C for 10–30 minutes with biotin-labeled secondary antibodies diluted in 1% BSA-PBS. The slices were then rinsed three times in PBS before being incubated in SABC for 10–30 minutes at 37°C and then rinsed three times in PBS. Labeled proteins were visualized by incubation in 50–100 *μ*L DAB substrate at the temperature of 15–25°C for 10 min, protected from light. Sections were counterstained with hematoxylin, PBS-rinsed, gradient alcohol dehydrated, cleared in xylene, and sealed with a neutral gum. Brown color staining was considered positive. Light microscopy was performed on 500 cells. Positive cells with less than 5% were classified as negative “−,” 5%–25% as weakly positive “+,” 25%–50% as moderately positive “++,” and >50 percent as significantly positive “+++,” according to the scoring system.

### 2.7. Protein Extraction and Western Blot Analysis

Rat liver tissues were isolated and total protein was extracted with a RIPA buffer (AR0105, BOSTER, CA, USA) with protease inhibitors (AR1182, BOSTER, CA, USA). The BCA method was applied to determine protein concentrations, and the groups of samples were regulated to the same concentration using ultrapure water. After electrophoresis, 50 g of protein from each sample was submitted to SDS-PAGE and transmitted to a PVDF membrane. Membranes were incubated with the primary antibody (1 : 1,000) and incubated overnight at 4°C. The next day, the membrane was bathed with TBS-T and a HRP-labeled goat anti-rabbit secondary antibody (1 : 5,000) was supplemented at 18–20°C for 60 min. After washing with TBS-T, protein bands were visualized by adding a luminescent solution for color development. The bands were converted into grayscale values using the Image J computer program, and the relative protein expression was reflected by the grayscale value of the target band and normalized to *β*-actin. To ensure reproducibility, the aforementioned tests were repeated at least three times.

The following antibodies were used: Bcl-2 (AF6139, 1 : 1000, AFFITY), Bax (A00183, 1 : 1000, BOSTER), p38MAPK (8690T, 1 : 1000, CST), p-p38MAPK (4511T, 1 : 1000, CST), caspase-3 (BA2142, 1 : 1000, BOSTER), *β*-actin (BM0627,1 : 2000, BOSTER), and GAPDH (BM1623, 1 : 2000, BOSTER). The secondary antibody was purchased from BOSTER (BA1054, BA1050, 1 : 5000).

### 2.8. Statistics

All the experiments were repeated three times, and the results were provided as mean ± SD. Prism statistical computer program (GraphPad8.0 software, US) was applied in the statistical analysis. One-way ANOVA was applied to compare the quantitative data from multiple groups, and statistical significance was considered when *P* < 0.05.

## 3. Results

### 3.1. Changes in General Morphology, Survival Rate, and Body Weight of Rats in Each Group

#### 3.1.1. General Changes and Survival Rate of the Rats

**Figure 1:**
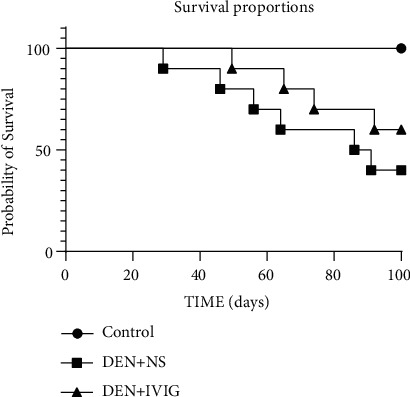
Difference in the survival rate of rats in each group during the experiment.

At 14 weeks after initiation of medication, the rats in the control group had smooth, white hair, normal weight gain, normal urine and stool, and none of the rats in the control group died, resulting in a 100% survival rate (Figure 1). Rats in the DEN + NS group had lusterless, rough, loose, disorganized, waxy and yellow hair, and hair loss. Some rats in this group died before the 14th week of the experiment, with a survival rate of 40% (Figure 1). Before death, they were depressed, ate and drank poorly, reduced activity, had serious weight loss, and started to get cranky. Rats in the DEN + IVIG group had coarser and more disorganized hair, lighter color waxing, and hair loss in comparison to the control group, along with fair weight gain and activity, a slightly sluggish reaction, slightly poorer spirit, diarrhea, and thinner body size, compared to the rats in the control group. All the above changes were not serious as that in the DEN + NS group. Besides, the survival rate of rats in DEN + IVIG group was 60%, clearly distinct from the control group and the DEN + NS group (*P* < 0.01).

#### 3.1.2. Body Weight Change in Rats

Before the experiment, there was no distinction in body weight of rats in the three groups (*P* > 0.05). The changes in body mass of rats were recorded weekly during the experimental period. By analyzing the data at different observation points (weeks 10, 12, and 14), we found that the body mass of rats in the control group increased steadily, indicating that they are in good health condition and normal growth. In contrast, the body weight of rats in both the DEN + NS and DEN + IVIG groups showed a tendency to decrease. Weight loss was statistically significant in the DEN + NS group at 10, 12 and 14 weeks, as well as the DEN + IVIG group at >12 weeks (*P* < 0.05). There was a statistical significant trend toward higher body weight in the DEN + IVIG group as compared to the DEN + NS group(*P* < 0.05, Table 1).

### 3.2. Liver Surface Changes and Assessment of HCC

**Figure 2:**
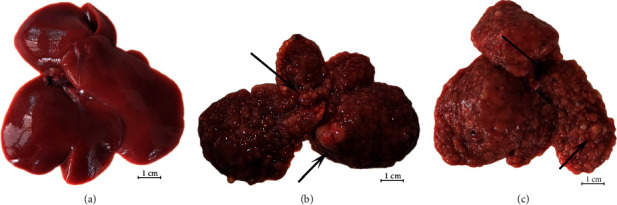
Representative images of rat livers from each group. Black arrows indicate tumors.

The liver and blood of rats were sampled and tested 14 weeks after DEN and drug injection. Morphological observation of the liver of each group showed that the control group displayed normal appearance, with a smooth surface, red color, soft texture and sharp liver edges, and none of the rats had hyperplastic nodules or hepatocellular carcinoma (Figure 2(a)). However, the liver in the DEN + NS group was highly fibrotic with uneven margins, dark or pale in color, and the liver surface was rough. Scattered grayish-white cancer nodules of different sizes were found. Some nodules fused together to form a giant mass (Figure 2(b)), with necrosis and hemorrhage on the cut surface, and bloody ascites in 60% of the DEN + NS rats. White hepatocellular carcinoma nodules were also seen in the livers of DEN + IVIG rats (Figure 2(c)), but the nodules were relatively smaller and less numerous than those in the DEN + NS group, and none of the DEN + IVIG rats displayed bloody ascites, invasion to the lungs or abdominal metastases on autopsy.

### 3.3. Liver Pathology in Rats

**Figure 3:**
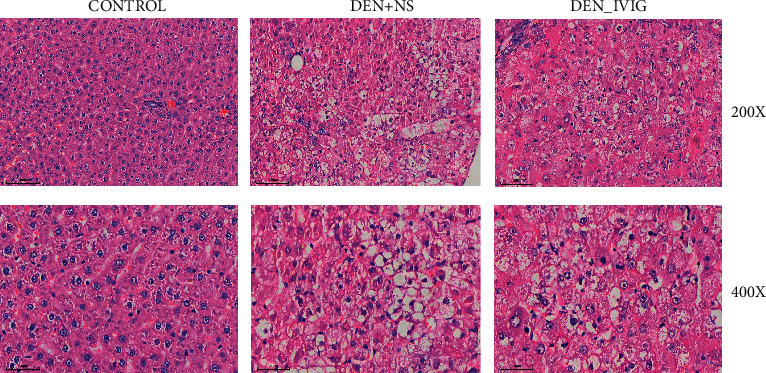
Representative H&E staining of the rat liver from each group. The scale bar represents 100 *μ*m in the low magnification field of view and 50 *μ*m in the high magnification field of view, *n* = 20.

In Figure 3, the hepatic tissue of control rats was normal in structure, with neatly arranged hepatocyte cords and regular surroundings, and endothelial cells arranged in the hepatic sinusoids had intact cell membranes and clear nuclei. Compared with the control group, the DEN + NS rats showed tumors, loss of normal hepatocyte structure, widening of hepatic cords in the liver parenchyma, polymorphic cells, increased nuclear division and cytoplasmic secretory granules, disorganized hepatocyte cords, large nucleus/cytoplasmic ratio, deep nuclear staining, and grade III cancer lesions. Compared with the DEN + NS group, the DEN + IVIG rats displayed less degenerative necrosis and lower malignancy of hepatocytes, showing focal necrosis and less nuclear schizophrenia, with some areas showing lamellar necrosis and grade II-III cancer lesions (Figure 3).

### 3.4. Serum Level of ALT, AST, ALP, and GGT

**Figure 4:**
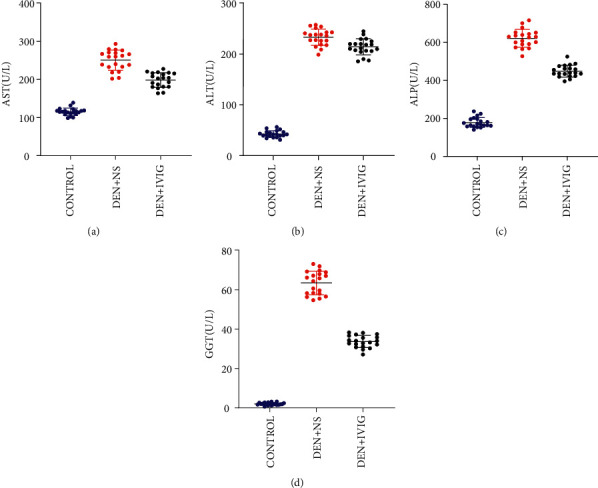
(a) The serum AST level. (b) The serum ALT level. (c) The serum ALP level. (d) The serum GGT  expression level. Values are given as the mean ± SD.

Liver function indexes collected from rats at week 14 showed that the level of AST, ALT, ALP, and GGT of rats in the DEN + NS group were higher than those in the control group (*P* < 0.05), and the liver function indexes in the DEN + IVIG group were lower than those in the DEN + NS group (*P* < 0.05).  Table 2 and Figure 4 show the effect of IVIG on AST, ALT, ALP, and GGT on DEN-induced HCC in rats.

### 3.5. Immunohistochemistry

**Figure 5:**
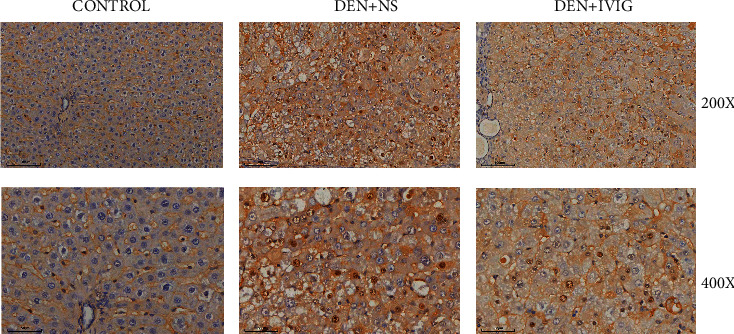
Representative images of immunohistochemical staining for c-myc-positive cells in rat liver tissues. The presence of brownish-yellow staining in the nuclei of the cells is considered as positive cells. The scale bar represents 100 *μ*m in the low magnification field of view and 50 *μ*m in the high magnification field of view.

As observed by immunohistochemical staining of c-myc in the rat liver tissue, c-myc-positive cells were brownish-yellow in color, expressed in the nucleus, and mainly distributed in cancer foci. The expression of c-myc was enhanced in the DEN + NS group and the DEN + IVIG group when compared to the control group (*P* < 0.05). The level of c-myc was reduced in the DEN + IVIG group in comparison to the DEN + NS group (*P* < 0.05) (Figure 5, Table 3).

### 3.6. p38MAPK Signaling Pathway and Apoptosis-Related Proteins

#### 3.6.1. Effect of IVIG on p38MAPK and p-p38MAPK

**Figure 6:**
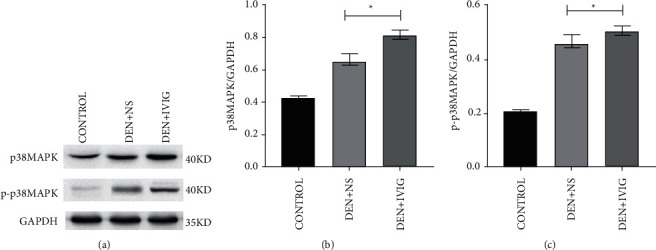
Western blotting on the p38MAPK and p-p38MAPK levels in rats' liver tissues in each group at the conclusion of the experiment. Quantitative analysis was performed with Image J software (NIH). ^*∗*^Significant differences (*P* < 0.05) were observed compared with the DEN + NS group.

The results of the effect of IVIG on total p38MAPK protein and phosphorylated p38MAPK (p-p38MAPK) protein in the rat liver are shown in Figure 6. The total p38MAPK and p-p38MAPK in the livers of rats in the DEN + IVIG group were 1.51− and 2.49−fold higher than that of the control, respectively, (*P* < 0.05). In contrast to the DEN + IVIG group, the liver tissues in the DEN + NS group had lower levels of p38MAPK and p-p38MAPK protein expressions, with the expression levels reduced by 15.2% and 7.6%, respectively (*P* < 0.05).

#### 3.6.2. Effect of IVIG on Bcl-2 and Bax

**Figure 7:**
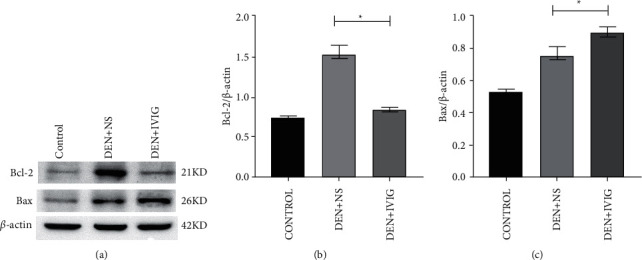
Western blotting detection of Bax and Bcl-2 protein expressions in rats' liver tissues in each group at the end of the experiment. Band intensities were quantified by Image J software (NIH). ^*∗*^Significant difference (*P* < 0.05) was observed in contrast with the DEN + NS group.

To clarify the regulatory mechanism of IVIG-induced apoptosis in hepatocellular carcinoma cells, we detected Bcl-2 and Bax levels in the rat livers (Figure 7). Under the carcinogenic effect of DEN, the protein content of Bcl-2 in the DEN + NS and DEN + IVIG groups increased 2.12- and 1.21-fold, respectively, in comparison to that in the control rats. The Bcl-2 in the DEN + IVIG group reduced by 43.1% by comparison with the DEN + NS group (*P* < 0.05). The Bax protein expression in rat liver was increased by IVIG treatment, and the expression level in the DEN + IVIG group was 1.17-times higher than that in the DEN + NS group, with the Bax protein content in the DEN + IVIG group being enhanced by 14.4% compared with the DEN + NS group (*P* < 0.05).

#### 3.6.3. Effect of IVIG on Apoptosis-Related Protein Cleaved Caspase-3

**Figure 8:**
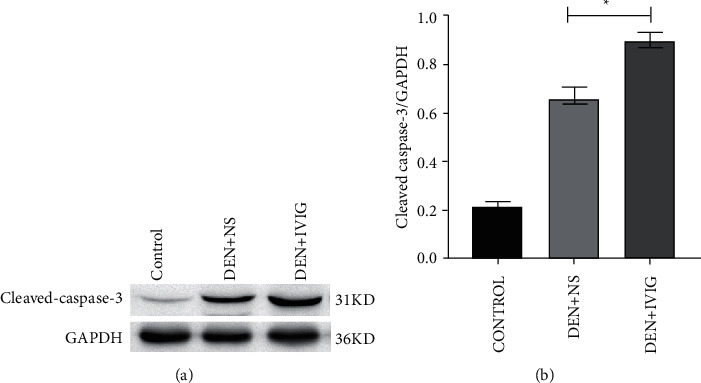
Western blotting on cleaved caspase-3 protein levels in liver tissues of rats in each group at the conclusion of the experiment. ^*∗*^Significant difference (*P* < 0.05) was observed in comparison with the DEN + NS group.

The expression level of cleaved caspase-3 in the livers of rats in each group is shown in Figure 8. In comparison to the DEN + NS group, the expression level of cleaved caspase-3 protein was typically elevated in the DEN + IVIG group and was 1.38-times higher than that in the DEN + NS group, with an increase of 27.5% (*P* < 0.05).

Taken together, in comparison with the control group, expressions of p38MAPK and p-p38MAPK proteins were typically upregulated in the DEN + IVIG and DEN + NS groups, and the expression level of p38MAPK and p-p38MAPK proteins in liver tissues was lower in the DEN + NS group in comparison to the DEN + IVIG group (*P* < 0.05). The cleaved caspase-3 and Bax protein expression was enhanced and Bcl-2 protein expression was weakened in the DEN + IVIG group in comparison to the DEN + NS group, while the Bax/Bcl-2 ratio increased (*P* < 0.05). All the abovementioned results suggest that IVIG intervention can induce apoptosis and delay the progression of hepatocellular carcinoma in rats.

## 4. Discussion

Hepatocarcinogenesis involves a malignant transformation process with multiple etiologies and multiple signaling pathways. Currently, therapeutic strategies targeting various signaling pathways have received widespread attention for the development of anticancer drugs [8]. In this study, DEN 200 mg/kg intraperitoneal injection was used to induce primary liver cancer, followed by 0.05% of DEN aqueous solution drinking ad libitum. After 12 weeks of DEN exposure, the survival rate and body mass of animals in the DEN + IVIG group were higher than those in the DEN + NS group. The nodules in the liver were massive, with necrosis and hemorrhage on the cut surface in the DEN + NS group, while the nodules in the DEN + IVIG group were relatively smaller and less numerous. These results suggest that IVIG can reduce the development of DEN-induced hepatocellular carcinoma and has a delaying effect on the progression of hepatocellular carcinoma in rats.

ALT and AST are sensitive indicators of direct hepatocyte damage. When ALT and AST are abnormally elevated, it indicates necrotic damage of hepatocytes [9]. ALP, as a functional liver enzyme, is strongly tied to lipid membranes in bile ducts, and its elevation indicates pathological bile flow problems [10]. GGT is a membrane-bound enzyme with tissue-specific expression and cancer-related alterations [11]. In this study, we found that the values of ALT, AST, ALP, and GGT in the DEN + IVIG group were substantially lower than those in the DEN + NS group, indicating that the use of IVIG protected the liver cells from damage. The pathological change in the liver of each group of rats was observed by HE staining and found that carcinogenesis was successfully induced in the DEN + NS group [12]. The hepatocyte hyperplasia, degeneration, hyperchromatic staining of nuclei, high proliferation, and a few venous cancer thrombi were seen at 14 weeks after induction of hepatocarcinogenesis. In the DEN + IVIG group, the lobular structure of the liver showed punctate and focal necrosis, the nuclear heterogeneity of the cells was greater, and the pathological changes of induced cancer were reduced compared with the DEN + NS group. This result suggests that IVIG was able to inhibit the carcinogenic process of DEN to some extent and protected the liver. IVIG can effectively inhibit tumor proliferation, infiltration, and metastasis in tumor cell lines, tumor animal models, and tumor patients [5, 6], such as oral squamous carcinoma [13], renal cell carcinoma [14], and breast cancer [15], but the antitumor mechanism of action of IVIG is not fully understood.

The occurrence of hepatocellular carcinoma is a complicated process involving numerous factors and multiple pathways. Many signaling pathways, such as the tyrosine kinase, PI3K/Akt, MAPK, Wnt/p-Catenin, and Hedgehog signaling pathways, have been found to be engaged in the formation of hepatocellular carcinoma [16, 17]. Among them, the mitogen-activated protein kinase (MAPK) signaling pathways play an important role [18, 19]. The MAPK signaling pathway is engaged in the regulation of cell growth, apoptosis, differentiation, and proliferation [20]. The p38MAPK signaling pathway, as an important member of the MAPK family, has been demonstrated to regulate apoptosis by activating Bax translocation, enhancing caspase-3 activation, elevating c-myc expression, and phosphorylating p53 [21, 22], which has not been reported in IVIG-related studies. IVIG administration during hepatocellular carcinoma induction in rats, the p38MAPK pathway was activated in the DEN + IVIG group, and the expression level of p38MAPK and p-p38MAPK was enhanced compared with the DEN + NS group and control group. This finding reveals that the MAPK signaling pathway was engaged in the regulation of IVIG-induced apoptosis in hepatocellular carcinoma cells. To further clarify the regulatory mechanism of IVIG-caused apoptosis in hepatocellular carcinoma cells, we evaluated the expression of two vital members from the Bcl-2 family, Bcl-2 and Bax, and the executioner caspase, cleaved caspase-3, at the protein level by Western blotting.

In the regulation of cell apoptosis, the Bcl-2 protein family plays a key role. Two proteins of the Bcl-2 protein family, antiapoptotic and proapoptotic, have opposite regulatory effects under either physiological or pathological conditions and are closely associated with hepatocarcinogenesis [23]. During hepatocellular carcinoma induction, the expression of Bcl-2, a downstream target gene of p38MAPK, was decreased, disrupting the balance between Bax and Bcl-2, and leading to the relative overexpression of Bax, ultimately causing apoptosis in hepatocellular carcinoma cells. Therefore, the apoptosis of hepatocellular carcinoma cells was substantially enhanced in the DEN + IVIG group in contrast to the DEN + NS group. In addition, the Bcl-2 family indirectly controls cell-autonomous death by interacting with the proto-oncogene c-myc [24].

C-myc is a nuclear protein transcription key molecule that acts a prominent role in cell growth and differentiation, cell proliferation, and apoptosis [25–27] and is aberrantly expressed in human hepatocellular carcinoma [28]. Our immunohistochemical results showed that c-myc was detected at a lower level in the control group, but significantly enhanced in DEN-induced rat liver tissues, particularly in the cancerous foci. IVIG intervention caused reduced c-myc expression, suggesting that the mechanism of action of IVIG may be achieved by inhibiting the expression of oncogenes to induce apoptosis in rat hepatocellular carcinoma cells, thereby inhibiting the progression of hepatocellular carcinoma.

## 5. Conclusions

In conclusion, our study highlights the significance of IVIG as an effective treatment to alleviate the progression of DEN-induced hepatocellular carcinoma. IVIG reduces the development of hepatocellular carcinoma in rats and improves liver function and protects against the hepatotoxic effects caused by DEN. IVIG regulates apoptosis in hepatocellular carcinoma cells by altering the expression level of apoptosis-related proteins in the rat liver. The mechanism may involve IVIG-mediated activation of the p38-MAPK signaling pathway, which influences the expression of related target proteins downstream of the pathway. Our findings could lead to a new strategy to reduce the damage caused by HCC progression.

## Figures and Tables

**Table 1:** Changes in the body weight of rats (*x* ± *s*, g).

Group	10 weeks	12 weeks	14 weeks
Control	424.12 ± 37.2	467.25 ± 35.2	501.20 ± 7.3
DEN + NS	360.05 ± 19.1^*∗*^	350.23 ± 18.2^*∗*^	340.21 ± 15.7^*∗*^
DEN + IVIG	402.56 ± 6.4^#^^*∗*^	390.41 ± 31.3^#^^*∗*^	378.07 ± 36.6^#^^*∗*^

^*∗*^clearly distinct from the control group (*P* < 0.05). ^#^ clearly distinct from the DEN + NS group (*P* < 0.05).

**Table 2:** Effect of IVIG on AST, ALT, ALP, and GGT on DEN-induced HCC in rats.

Group treatment	AST (*μ*/L)	ALT (*μ*/L)	ALP (*μ*/L)	GGT (*μ*/L)
CONTROL	114.75 ± 7.68	41.87 ± 2.90	178.13 ± 26.00	2.02 ± 0.66
DEN + IVIG	198.62 ± 19.36^*∗*^^#^	214.25 ± 17.93^*∗*^^#^	448.38 ± 31.25^*∗*^^#^	33.75 ± 3.06^*∗*^^#^
DEN + NS	250.75 ± 26.34^*∗*^	232.13 ± 18.76^*∗*^	621.00 ± 46.73	63.88 ± 7.04^*∗*^

Values are given in the form of means ± SD. (*x* ± *s*, *n* = 20). ^*∗*^clearly distinct from the control group (*P* < 0.01). ^#^ clearly distinct from the DEN + NS group (*P* < 0.01).

**Table 3:** Expression of c-myc in liver tissues of various groups of rats with HCC.

Group	*N*	c-myc expression				Expression rate %
		−	+	++	+++	
Control	20	18	2	0	0	10.0
DEN + NS	20	3	14	3	0	85.0^*∗*^
DEN + IVIG	20	9	8	2	1	55.0^*∗*^^#^

*N* represents the number of rats of each group. ^#^ clearly distinct from the DEN + NS group (*P* < 0.05). ^*∗*^clearly distinct from the control group (*P* < 0.05).

## Data Availability

The data can be obtained upon request from the corresponding author via e-mail(12xmliu2@stu.edu.cn).
